# Diagnostic Performance of Magnetic Resonance Enterography Disease Activity Indices Compared with a Histological Reference Standard for Adult Terminal Ileal Crohn’s Disease: Experience from the METRIC Trial

**DOI:** 10.1093/ecco-jcc/jjac062

**Published:** 2022-06-08

**Authors:** Shankar Kumar, Thomas Parry, Sue Mallett, Gauraang Bhatnagar, Andrew Plumb, Shaun Walsh, Nigel Scott, Ruchi Tandon, Heung Chong, John du Parcq, Adrianna Martinez, Morgan Moorghen, Manuel Rodriguez-Justo, Steve Halligan, Stuart A Taylor, METRIC study investigators, Rachel Baldwin-Cleland, Stuart Bloom, Arun Gupta, Peter J Hamlin, Ailsa L Hart, Antony Higginson, Ilan Jacobs, Sara McCartney, Anne Miles, Charles D Murray, Richard C Pollok, Shonit Punwani, Laura Quinn, Zainib Shabir, Andrew Slater, Damian Tolan, Simon Travis, Alastair Windsor, Peter Wylie, Ian Zealley, Jade Dyer, Pranitha Veeramalla, Sue Tebbs, Steve Hibbert, Richard Ellis, Fergus Thursby-Pelham, Richard Beable, Nicola Gibbons, Claire Ward, Anthony O’Connor, Hannah Lambie, Rachel Hyland, Roger Lapham, Doris Quartey, Deborah Scrimshaw, Helen Bungay, Maggie Betts, Simona Fourie, Niall Power, Rajapandian Ilangovan, Uday Patel, Evgenia Mainta, Phillip Lung, Ian Johnston, Mani Naghibi, Francois Porte, Christopher Alexakis, James Pilcher, Anisur Rahman, Jonny Vlahos, Rebecca Greenhalgh, Anita Wale, Teresita Beeston, Wivijin Piga, Joey Clemente, Farooq Rahman, Simona de Caro, Shameer Mehta, Roser Vega, Roman Jastrub, Harbir Sidhu, Hameed Rafiee, Mairead Tennent, Caron Innes, Craig Mowat, Gillian Duncan, Steve Morris

**Affiliations:** Centre for Medical Imaging, University College London, London, UK; Centre for Medical Imaging, University College London, London, UK; Centre for Medical Imaging, University College London, London, UK; Centre for Medical Imaging, University College London, London, UK; Centre for Medical Imaging, University College London, London, UK; Department of Pathology, Ninewells Hospital and Medical School, Dundee, UK; Department of Histopathology, Leeds Teaching Hospitals NHS Trust, Leeds, UK; Department of Cellular Pathology, Oxford University Hospitals NHS Foundation Trust, Oxford, UK; Department of Cellular Pathology, St George’s University Hospitals NHS Foundation Trust, London, UK; Department of Cellular Pathology, St George’s University Hospitals NHS Foundation Trust, London, UK; Department of Cellular Pathology, St Mark’s Hospital, London North West University Healthcare NHS Trust, London, UK; Department of Cellular Pathology, St Mark’s Hospital, London North West University Healthcare NHS Trust, London, UK; Department of Histopathology, University College Hospital NHS Foundation Trust, London, UK; Centre for Medical Imaging, University College London, London, UK; Centre for Medical Imaging, University College London, London, UK

**Keywords:** Crohn’s disease, imaging, magnetic resonance enterography

## Abstract

**Background and Aims:**

The simplified magnetic resonance enterography [MRE] index of activity [sMARIA], London, and ‘extended’ London, scoring systems are widely used in Crohn’s disease [CD] to assess disease activity, although validation studies have usually been single-centre, retrospective, and/or used few readers. Here, we evaluated these MRE indices within a prospective, multicentre, multireader, diagnostic accuracy trial.

**Methods:**

A subset of participants [newly diagnosed or suspected of relapse] recruited to the METRIC trial with available terminal ileal [TI] biopsies was included. Using pre-specified thresholds, the sensitivity and specificity of sMARIA, London, and ‘extended’ London scores for active and severe [sMARIA] TI CD were calculated using different thresholds for the histological activity index [HAI].

**Results:**

We studied 111 patients [median age 29 years, interquartile range 21-41, 75 newly diagnosed, 36 suspected relapse] from seven centres, of whom 22 had no active TI CD [HAI = 0], 39 mild [HAI = 1], 13 moderate [HAI = 2], and 37 severe CD activity [HAI = 3]. In total, 26 radiologists prospectively scored MRE datasets as per their usual clinical practice. Sensitivity and specificity for active disease [HAI >0] were 83% [95% confidence interval 74% to 90%] and 41% [23% to 61%] for sMARIA, 76% [67% to 84%] and 64% [43% to 80%] for the London score, and 81% [72% to 88%] and 41% [23% to 61%] for the ‘extended’ London score, respectively. The sMARIA had 84% [69-92%] sensitivity and 53% [41-64%] specificity for severe CD.

**Conclusions:**

When tested at their proposed cut-offs in a real-world setting, sMARIA, London, and ‘extended’ London indices achieve high sensitivity for active TI disease against a histological reference standard, but specificity is low.

## 1. Introduction

Crohn’s disease [CD] is characterised by intermittent enteric inflammation, usually affecting the terminal ileum [TI].^1,2^ Accurate detection of active inflammation is pivotal to treat symptoms and improve long-term outcomes, reducing subsequent fistulae, strictures, and bowel resection.^3^ Endoscopy is the reference standard for diagnosis, but its invasive nature precludes repeated assessment and, furthermore, much of the small bowel is inaccessible unless capsule endoscopy is adopted.^4,5^ Magnetic resonance enterography [MRE] is a well-tolerated, radiation-free alternative also able to assess disease activity.^6–8^ Various MR activity scores that quantify disease activity have been validated, including the ‘magnetic resonance index of activity’ [MARIA], the London, and the ‘extended’ London indices.^9–11^ Although MARIA is reliable for measuring disease activity with high reproducibility, it is limited to the research setting because derivation is time-consuming and involves two quantitative variables.^4,12^ The recently developed simplified MARIA [sMARIA] addresses this and, like the London and ‘extended’ London indices, does not require quantitative calculation.^13,14^ These three scoring systems are therefore potentially better suited for routine clinical practice than MARIA, but have been largely derived and validated via retrospective, single-centre studies with few readers, so their generalisability is uncertain.^11,13,14^ This study aimed to compare the sMARIA, London, and ‘extended’ London indices prospectively for quantifying terminal ileal CD activity using a histopathological reference standard acquired during a multicentre, multireader, diagnostic accuracy trial.^15^

## 2. Materials and Methods

### 2.1. Study population

We studied a subset of patients recruited to the MR Enterography or Ultrasound in Crohn’s disease [METRIC] trial.^15,16^ Briefly, METRIC was a multicentre, non-randomised, single-arm, prospective, diagnostic accuracy study comparing MRE and enteric ultrasound [US] for the presence, extent, and activity of small bowel CD. To reflect routine clinical practice, patients with newly diagnosed CD or established CD with suspected relapse were recruited from eight UK National Health Service [NHS] hospitals, all with a well-established MRE service. All patients underwent MRE and enteric US in addition to other investigations, such as endoscopy, as part of usual clinical care. Patients were eligible if aged ≥16 years, had no contraindications to MRE, and were not pregnant. Patient demographic and clinical data including age, sex, medication, Harvey-Bradshaw index, C-reactive protein [CRP] concentration, faecal calprotectin concentration, and EuroQol [EQ]-5D score were collated by the trial team.

### 2.2. Study design

**Figure 1.**
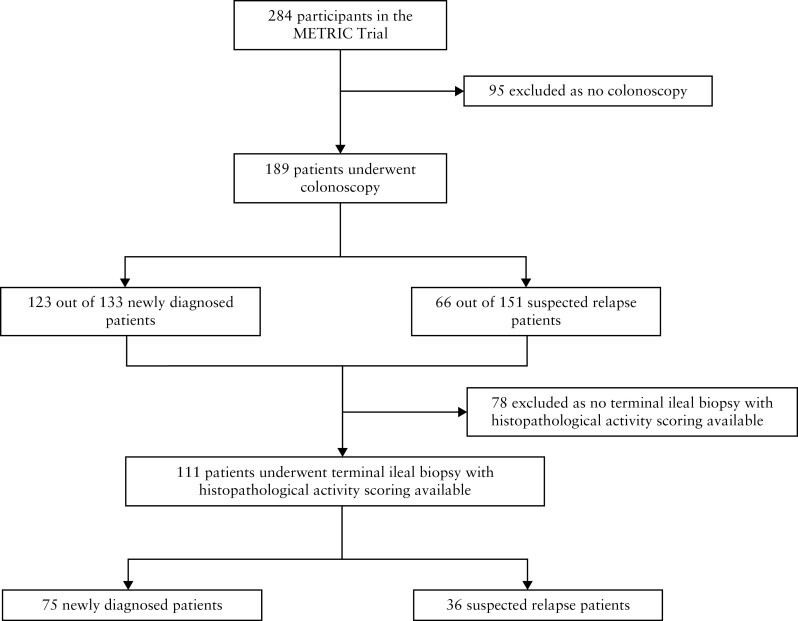
Flow chart of the study population.

The METRIC protocol stipulated colonoscopy if part of usual clinical care [rather than a research intervention], although all newly diagnosed patients had to have already undergone colonoscopy or had this planned at the time of recruitment. For the present study, we identified all patients [both suspected and established CD] with available terminal ileal biopsy within 4 weeks of MRE, and with histological activity scoring [Figure 1]. The study was reported according to TRIPOD reporting guidelines for validation studies.^17^

### 2.3. MRE protocol

MRE was performed according to local protocols on either 1.5 or 3 Tesla platforms with a minimum number of data sequences acquired: coronal steady-state free precession gradient echo [SSFP GE] sequences without fat saturation, hyoscine butylbromide 20 mg intravenously [IV], axial and coronal fast spin echo [FSE] T2-weighted [T2W] sequences without fat saturation, coronal FSE T2W sequences with fat saturation, axial diffusion-weighted images [b values 50 and 600], and unenhanced coronal T1W sequences with fat saturation followed by contrast-enhanced T1W sequences with fat saturation [60-70 s post-injection].^15^ At each site, local radiologists who were Fellows of the Royal College of Radiologists and had at least 1 year of subspecialty training in gastrointestinal radiology interpreted MRE. Radiologists were blind to all contemporaneous imaging and clinical data, including endoscopy, and completed a standardised clinical report form [CRF] prospectively, documenting conventional MRE observations [Supplementary Appendix 1], which we used to derive the sMARIA,^13^ London,^11^ and ‘extended’ London^11^ activity scores [see below].

### 2.4. Histopathological reference standard

Terminal ileum biopsies were scored by a specialised gastrointestinal histopathologist at each individual site, using the histological activity index [HAI].^18^ They were blinded to the MRE findings. The specimen with the most severe inflammation was used to categorise patients into one of four groups: 0, remission; 1, mild activity; 2, moderate activity; 3, severe activity.

### 2.5. Derivation of MRE activity scores

The sMARIA,^13^ London,^11^ and ‘extended’ London^11^ activity scores were derived from the completed radiologist CRFs as follows:

sMARIA = [1 × wall thickness >3 mm] + [1 × wall oedema] + [1 × fat stranding] + [2 × ulcers].

London = 1.79 + [1.34 × mural thickness] + [0.94 × mural T2 score].

‘Extended’ London = mural thickness + mural T2 score + perimural T2 signal + contrast enhancement.

Full definitions for each of the MRE activity scores are provided in Supplementary Appendix 2.

We assessed the presence of wall oedema, a feature of sMARIA, using mural T2 signal, where a score of 1 or more was taken to represent the presence of oedema. Fat stranding on MRE, also a component of sMARIA, was assessed using a perimural T2 signal score. A score of 1 or more was considered to indicate fat stranding.

### 2.6. Statistical analysis

Study outcomes pre-specified analysis of diagnostic accuracy as sensitivity and specificity of MRE scoring systems for: [i] CD disease activity of the TI; and [ii] sMARIA for diagnosing severe TI CD. Diagnostic outcomes were analysed using histology as the reference standard and stratified by newly diagnosed and suspected relapsed patients.

We performed two analyses for study outcome [i], using two reference standard thresholds: HAI cut-off of 0 versus 1, 2, or 3 for active disease and an HAI cut-off of 0 or 1 versus 2 and 3 for active disease.

For outcome [ii], the reference standard was dichotomised into non-severe activity [normal/mild/moderate, HAI = 0, 1 or 2] and severe activity [severe, HAI = 3].

Based on the original development and validation studies for each score, thresholds for active CD were defined a priori as an sMARIA score >= 1,^13^ London score >= 4.1, and an ‘extended’ London score >= 3.^11^ Severe CD [HAI score = 3] was considered present if sMARIA was >= 2^13^ [equivalent thresholds are not available from London or ‘extended’ London scores]. There were no missing data for any outcome.

Diagnostic accuracy measures [sensitivity and specificity] were calculated for each scoring system with 95% confidence intervals [CI] around the pre-specified thresholds, presented within receiver operating characteristic [ROC] curves [STATA commands: rocfit and rocplot]. Statistical significance was based on Wilson’s 95% confidence intervals.^19^ Analyses were performed using Stata 16 [College Station, TX, USA].

### 2.7. Ethical considerations

Ethical approval was obtained in September 2013 [13/SC/0394], and patients provided written consent.

## 3. Results

### 3.1. Study population and patient characteristics

**Figure 2.**
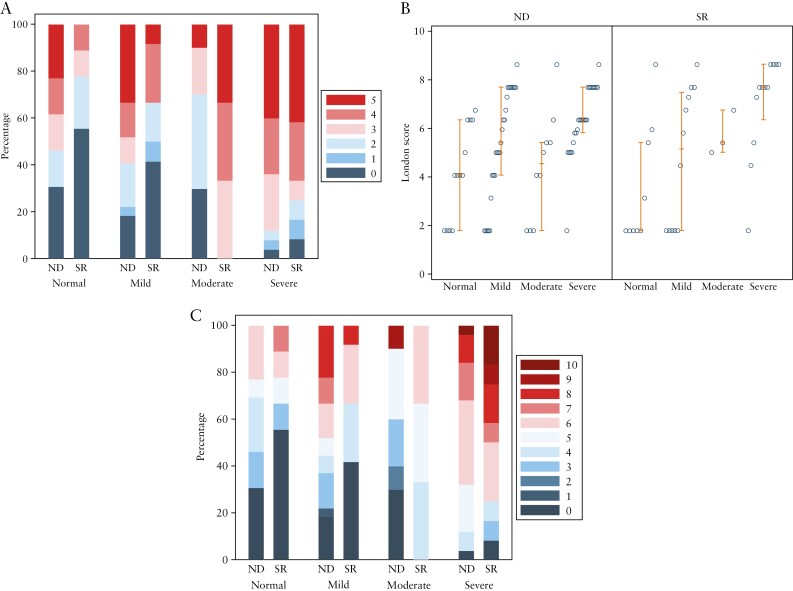
Distribution of the activity scores against HAI values stratified by newly diagnosed and suspected relapse patients. [a] sMARIA, [b] London, and [c] ‘extended’ London score. ND, newly diagnosed; SR, suspected relapse; HAI, histological activity index.

Of the 284 participants in METRIC, 189 underwent colonoscopy of whom 111 from seven institutions had available terminal ileal histopathological activity scores and were included in the present analysis. Of these 111, 75 patients were newly diagnosed and 36 had established CD [Figure 1]. MRE studies were read by one of 26 radiologists. Patient demographics, clinical characteristics, and the breakdown of HAI scores for each group are presented in Table 1. Figure 2 illustrates the spread of the activity scores against HAI values. Clinical characteristics for patients with normal TI histology [HAI = 0] were not significantly different when stratified by CD activity based upon the MRE activity indices [Supplementary Tables 6-8].

**Table 1.** Patient demographics, clinical characteristics, and the breakdown of HAI scores of the study population.

Variable	All patients [*n *= 111]	Newly diagnosed CD [*n* = 75]	Suspected relapse [*n* = 36]
Age [years]			
Median [IQR]	29 [21 to 41]	26 [21 to 40]	34 [27 to 43]
Sex,no. [%]			
Female	56 [50]	33 [44]	23 [64]
Male	55 [50]	42 [56]	13 [36]
Medication, no. [%]			
ASA	8 [7]	5 [7]	3 [8]
Steroid	26 [23]	22 [29]	4 [11]
Immunomodulator	19 [17]	6 [8]	13 [36]
Anti-TNF antibodies	11 [10]	3 [4]	8 [22]
HBI			
Median [IQR]	4 [2 to 6]	3.5 [1 to 6]	4.5 [2 to 9]
EQ-5D			
Median [IQR]	70 [50 to 80]	70 [50 to 82.5]	65 [40 to 80]
CRP [mg/L]			
Median [IQR]	9 [3 to 26]	11 [3 to 29]	6 [2 to 23]
Calprotectin [μg/g]			
Median [IQR]	437 [151 to 716]	466 [137 to 686]	405 [247 to 780]
History of surgery, no. [%]	20 [18]	6 [8]	14 [39]
MRI platform, no. [%]			
1.5 T	79 [71]	56 [75]	23[64]
3.0 T	32 [29]	19 [25]	13 [36]
HAI, no. [%]			
0	22 [20]	13 [17]	9 [25]
1	39 [35]	27 [36]	12 [33]
2	13 [12]	10 [13]	3 [9]
3	37 [33]	25 [34]	12 [33]

Missing data—HBI , 9; EQ-5D , 12; CRP , 10; calprotectin , 50.

CD , Crohn’s disease; IQR , interquartile range; HBI , Harvey-Bradshaw Index; EQ-5D , EuroQol five-dimension questionnaire; TNF , tumour necrosis factor; CRP , C-reactive protein; HAI , histological activity index; T , Tesla; ASA, aminosalicylate; MRI, magnetic resonance imaging.

### 3.2. Performance characteristics of MRE activity scores for identifying active disease

**Figure 3.**
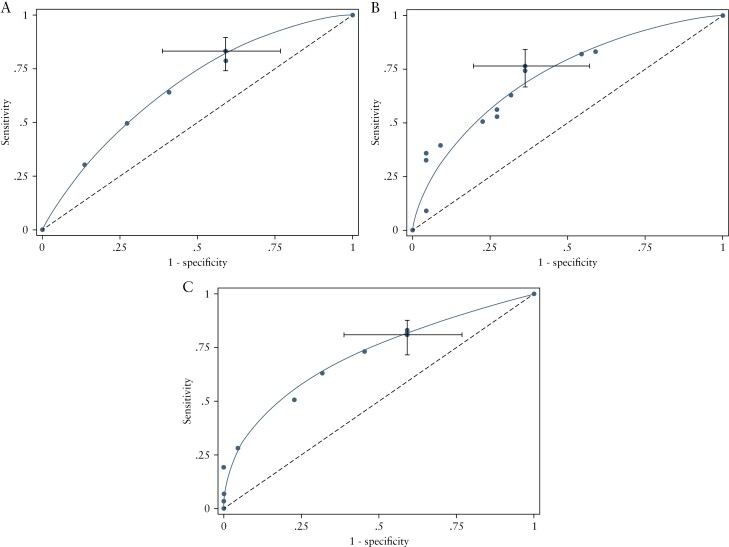
ROC curves for detecting active disease [HAI >0] for [a] sMARIA, [b] London, and [c] ‘extended’ London scores. Grey bars report 95% confidence intervals at the pre-specified thresholds. HAI, histological activity index; ROC, receiver operating characteristic.

**Figure 4.**
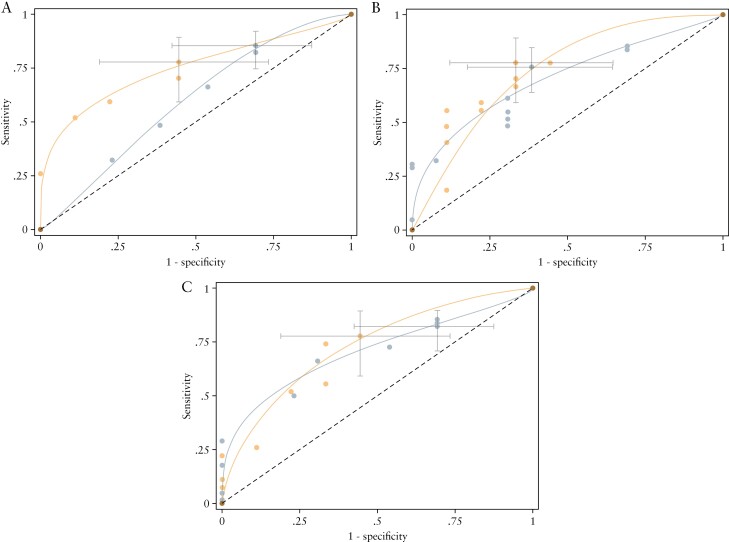
ROC curves for detecting active disease [HAI >0] stratified by newly diagnosed [grey] and suspected relapse [orange] patients for [a] sMARIA, [b] London, and [c] ‘extended’ London scores. Grey bars report 95% confidence intervals at the pre-specified thresholds. HAI, histological activity index; ROC, receiver operating characteristic.

Tables 2 and 3 detail the sensitivity and specificity with 95% CI of each MRE index for identifying active disease using previously defined thresholds for active disease defined as HAI >0 and HAI >1, respectively. Corresponding ROC curves when using the HAI >0 cut-off are presented in Figures 3 and 4 [ROC curves using HAI >1 to define active disease are provided in Supplementary Appendices 3 and 4]. Sensitivity was similarly high between all three scoring systems at pre-specified score thresholds. The London score had the highest specificity, but specificity was in general low for all three scores.

**Table 2.** Performance characteristics for the sMARIA, London, and ‘extended’ London activity scores for identifying active disease using HAI >0 to represent active disease; 95% confidence intervals reported.

sMARIA = simplified magnetic resonance enterography index of activity, HAI = histological activity index	**Cut-off**	**All patients [*n* = 111]**		Newly diagnosed [*n* = 75]		Suspected relapse [*n* = 36]	
		**Sensitivity**	**Specificity**	**Sensitivity**	**Specificity**	**Sensitivity**	**Specificity**
sMARIA	>=1	83 [74 to 90]	41 [23 to 61]	85 [75 to 92]	31 [13 to 58]	78 [59 to 89]	56 [27 to 81]
London	>=4.1	76 [67 to 84]	64 [43 to 80]	76 [64 to 85]	62 [36 to 82]	78 [59 to 89]	67 [35 to 88]
‘Extended’ London	>=3	81 [72 to 88]	41 [23 to 61]	82 [71 to 90]	31 [13 to 58]	78 [59 to 89]	56 [27 to 81]

**Table 3.** Performance characteristics for the sMARIA, London, and ‘extended’ London activity scores for identifying active disease using HAI >1 to represent active disease; 95% confidence intervals reported.

**Scoring System**	**Cut-off**	**All patients [n = 111]**		Newly diagnosed [n = 75]		Suspected relapse [n = 36]	
		**Sensitivity**	**Specificity**	**Sensitivity**	**Specificity**	**Sensitivity**	**Specificity**
sMARIA	>=1	90 [79 to 96]	31 [21 to 44]	89 [74 to 95]	23 [12 to 38]	93 [70 to 99]	48 [28 to 68]
London	>=4.1	86 [74 to 93]	46 [34 to 58]	83 [67 to 92]	43 [29 to 58]	93 [70 to 99]	52 [32 to 72]
‘Extended’ London	>=3	88 [76 to 94]	33 [22 to 45]	86 [71 to 94]	25 [14 to 40]	93 [70 to 99]	48 [28 to 68]

sMARIA, simplified magnetic resonance enterography index of activity; HAI, histological activity index.

For an HAI cut-off of >0 for active disease, in patients with a suspicion of relapsed disease, the 95% CI for specificity indicates that the discrimination of those without disease is above the diagonal line of 50% chance [Figure 4]. However, in newly diagnosed patients, the 95% CI for specificity at pre-specified thresholds for scores overlapped with chance, indicating that they did not help to discriminate patients without active disease. Bowel wall thickness was the most important parameter for identifying active disease; wall thickness above 3 mm was a dominant component of all scores in our patient cohort.

### 3.3. Performance characteristics of the sMARIA index for identifying severe disease

**Figure 5.**
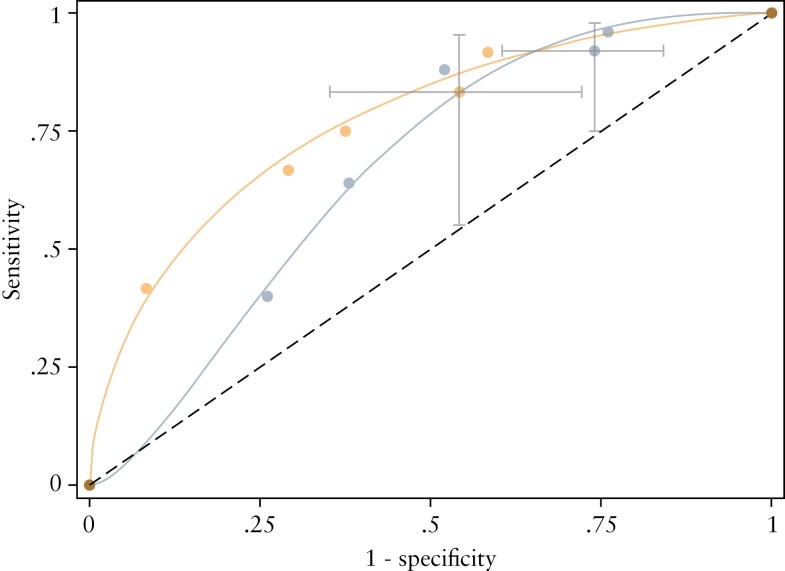
ROC curves for sMARIA for detecting severe disease stratified by newly diagnosed [grey] and suspected relapse [orange] patients. Grey bars report 95% confidence intervals at the pre-specified thresholds. ROC, receiver operating characteristic.

The accuracy of sMARIA for severe disease [=>2] is presented in Table 4, with the corresponding ROC in Figure 5. Sensitivity again exceeded specificity. Increased mural thickness was the most important factor to identify severe disease.

**Table 4.** Performance characteristics for sMARIA to identify severe Crohn’s disease; 95% confidence intervals reported.

**Scoring system**	**Cut-off**	**All patients [*n *= 111]**		Newly diagnosed [*n* = 75]		Suspected relapse [*n *= 36]	
		**Sensitivity**	**Specificity**	**Sensitivity**	**Specificity**	**Sensitivity**	**Specificity**
sMARIA	>=2	89 [75 to 96]	32 [23 to 44]	92 [75 to 98]	26 [16 to 40]	83 [55 to 95]	46 [28 to 65]

## 4. Discussion

We evaluated the accuracy of three validated MRE scoring systems via comparison against a histopathological reference standard as part of a generalisable, multicentre, multireader, prospective study. We found that all scoring systems were highly sensitive for detecting active CD of the TI. The London score had the highest specificity but, in general, specificity was low for all three scores. By way of illustration, for every 10 patients without TI histological activity [HAI = 0], on average between four and six patients would be incorrectly classified as having active TI CD based on the three MRI activity scores. Accuracy was greater in patients with relapsed disease compared with new diagnoses. Bowel wall thickness was the feature most associated with active and severe disease. The sMARIA index had high sensitivity for detecting severe TI disease [unavailable in the other scores] but low specificity.

MRE is an increasingly performed investigation that helps direct clinical decision making.^2,15,20–22^ There has been considerable interest in developing MRE activity scores that are objective, reproducible, quick to calculate, and not reliant upon gadolinium-enhanced sequences. In a single-centre study comprising a development cohort of 16 patients and validation cohort of 26 patients, with two reporting radiologists and a reference standard of endoscopic biopsy acute inflammatory score [eAIS, similar to the HAI score used in the current study], Steward *et al.* found that the London and ‘extended’ scores had a sensitivity of 81% [95% CI 54 to 96] and 87% [95% CI 61 to 98], and specificity of 70% [95% CI 35 to 93], respectively, for detecting active CD of the TI.^11^ In our diverse, multi-institution cohort with numerous readers, we found the London and ‘extended’ London scoring systems to be similarly sensitive [76% and 81%, respectively], but whereas the London score yielded a specificity of 64% [95% CI 43 to 80] using an HAI cut-off of 0, the specificity of the ‘extended’ London score was only 41% [95% CI 23 to 61]. Specificity was lower for all three scores when using an HAI cut-off of >1 for active disease. Puylaert *et al*. evaluated the London score in 98 patients from two tertiary referral centres, with two radiologists scoring the MRI studies,^23^ also limiting their study population to those with CD of the TI. When compared with the eAIS and Crohn’s Disease Index of Severity [CDEIS] reference standards, they found the London score had sensitivity of 79% [95% CI 67 to 89] and 82% [95% CI 69 to 91] and specificity of 63% [95% CI 46 to 77] and 71% [53 to 85], respectively, in identifying active CD. In our study, we noted similar performance characteristics, notwithstanding the multiple readers and centres and different reference standard.

The recently developed sMARIA, which consists of a simple dichotomic assessment of four variables, has been reported to be accurate for both identifying disease and assessing treatment response. Importantly, sMARIA is well suited to clinical practice because the time required for its calculation is significantly shorter than MARIA [4.5 min versus 12.4 min].^24^ In a single-centre study, Ordas *et al*. developed the sMARIA in a cohort of 98 patients, achieving a sensitivity and specificity of 90% and 81%, respectively for identifying active disease.^13^ Similar performance characteristics were reported for the detection of severe disease; sensitivity was 85% and specificity was found to be 92%. In another single centre, in a prospective series of 50 patients where ileocolonoscopy using the CDEIS was the reference standard, sMARIA detected active and severe disease with a sensitivity of 88.9% [95% CI 79.3 to 95.1] and 91.9% [95% CI 89 to 96.5], respectively, and specificity of 93.4% [95% CI 89 to 96.5] and 83.1% [95% CI 74.3 to 89.3], respectively.^14^ Roseira and colleagues evaluated the sMARIA index in a series of 84 patients.^25^ For identifying active disease, they reported that the per-segment diagnostic accuracy of sMARIA had a sensitivity and specificity of 90% and 98%, respectively. Similarly, Tao *et al.* found the sMARIA score provided a sensitivity of 81.4% and 88.9%, and a specificity of 90.8% and 95.1%, for identifying active and severe CD, respectively, with a Simple Endoscopic Score for Crohn’s Disease [SES-CD] reference standard.^26^ However, these two studies are limited by their retrospective nature, using data from single centres with only two readers, and this restricts generalisability greatly.

In the present study, we found sMARIA to have lower specificity for both active and severe CD. There are several possibilities to explain this. First, our study population comprised patients only with CD of the TI, so our results are expressed per segment, which cannot be directly compared with accuracy results per patient. Indeed, in the development cohort for sMARIA by Ordas *et al*., the calculations of sensitivity and specificity were based upon 495 bowel segments, of which 96 were of the TI and 399 were colonic.^13^ Similarly, the series by Capozzi *et al.* calculated the performance characteristics of sMARIA from 270 intestinal segments comprising 42 TI segments and 228 colonic segments.^14^ Our results likely reflect more accurately the performance characteristics for sMARIA when considering the TI exclusively. Second, we found wall thickness was the observation most associated with active disease and patients in whom disease is histologically quiescent may still exhibit bowel wall thickening, risking over-diagnosis. Third, the apparent low specificity may in part reflect the histopathological reference standard as well as true over-diagnosis using MRI. Endoscopic skipping is well described, where there is sparing of the luminal surface of the terminal ileum as the active inflammatory process is confined to intramural portions of the bowel wall, not visualisable at endoscopy. In this circumstance, MRE is more sensitive than biopsy.^27^ Indeed, Nehra *et al*. observed that 67% of individuals with a negative small bowel biopsy went on to have confirmation of disease progression; 70% of these patients demonstrated radiological worsening on subsequent studies, ulcer formation was later detected in 61% at ileocolonoscopy, and 68% required surgical resection.^28^ In our cohort of patients with normal histology of the TI [HAI = 0], the CRP and faecal calprotectin concentrations were numerically higher in the patients with active TI CD on MRE. This may reflect the limitations of a superficial histological reference standard versus transmural bowel wall assessment afforded by MRE. Statistical significance was not reached, although the sample size of this group was relatively small [*n* = 22] and likely underpowered. It is therefore possible that the apparent low specificity of the MRE activity scoring systems is due to the limitations of our reference standard, and this finding should be interpreted with caution.

The development and validation studies for the London, ‘extended’ London, and sMARIA scores took place in single centres with few readers and, in some cases, the MRE studies were read exclusively by radiologists with substantial inflammatory bowel disease [IBD] expertise.^11,13,14^ Our study is the first to compare the performance of these MRE activity scores with a histopathological reference standard in a prospective, multicentre, multireader setting. We examined 111 patients from seven institutions with 26 radiologists, a design we believe more likely to provide generalisable estimates than others, and approaching expected performance in clinical practice. Furthermore, to the best of our knowledge, ours is the largest sample in the literature to date with a good spread of patients with and without active disease. In our study, we found better accuracy was seen in patients with relapsed disease compared with those with a potential new diagnosis of CD. This may be explained by the fact that the development studies for all three MRE activity indices focused on patients with longstanding, established CD.

Our study has several limitations. First, despite adopting a robust histopathological reference standard, we could not use additional reference standards such as the Crohn’s Disease Endoscopic Index of Severity [CDEIS] or SES-CD, which means direct comparison with much of the current literature is limited. It is well established that endoscopically ‘normal’ mucosa can be inflamed on subsequent histology. Furthermore, an SES-CD score of between 0 and 2 is often used to define inactive disease, despite visible pathology and, by extension, implies histological inflammation.^27^ Second, we limited our analysis to the TI and did not evaluate the performance characteristics of the MRE indices in assessing colonic segments. Finally, METRIC was undertaken before sMARIA was developed. Therefore, fat stranding on MRE, a variable restricted to sMARIA, was not evaluated by us. We used perimural T2 signal as a surrogate for fat stranding, which we considered to be a reasonable alternative, but future studies ought to evaluate fat stranding specifically when calculating sMARIA. Similarly, we used mural T2 signal to assess for the presence of wall oedema, which we felt was an acceptable alternative.

In conclusion, this study provides evidence that the sMARIA, London, and ‘extended’ London indices are sensitive for detecting active CD of the TI in a real-world setting. Specificity is low for all three scores, with the London score having the highest. This in part may reflect the limitations of a histological standard of reference, as well as the fact that other studies have expressed per segment results, rather than per patient. Further prospective, multicentre, multireader studies with a range of robust reference standards are needed to help determine if the sMARIA, London, and ‘extended’ London indices can be adopted into routine clinical practice.

The data underlying this article will be shared on reasonable request to the corresponding author.

## Supplementary Material

jjac062_suppl_Supplementary_Appendix_1

jjac062_suppl_Supplementary_Appendix_2

jjac062_suppl_Supplementary_Appendix_3a

jjac062_suppl_Supplementary_Appendix_3b

jjac062_suppl_Supplementary_Appendix_3c

jjac062_suppl_Supplementary_Appendix_4a

jjac062_suppl_Supplementary_Appendix_4b

jjac062_suppl_Supplementary_Appendix_4c

jjac062_suppl_Supplementary_Tables
