# Supplementary material for: Diagnostic Performance of Magnetic Resonance Enterography Disease Activity Indices Compared with a Histological Reference Standard for Adult Terminal Ileal Crohn’s Disease: Experience from the METRIC Trial
Source: J Crohns Colitis. 2022 Jun 8;16(10):1531–9. doi: 10.1093/ecco-jcc/jjac062 (PMC9624291; doi:10.1093/ecco-jcc/jjac062)
Supplement: jjac062_suppl_Supplementary_Appendix_1 [file jjac062_suppl_supplementary_appendix_1.pdf]

METRIC

Site | | | | | | | | | | | | | | | Date of form | | | / | | | | / | | | | |

Patient Number | | | | | | Initials | | | | Date of birth | | | / | | | | / | | | | |

PATIENT CASE REPORT FORM: MRI INTERPRETATION

Radiologist Initials______________

MRI platform______________________

Central review (due to unblinding at recruitment site) Y/N

Are you blinded to other clinical tests and investigations and patient clinical history (other than previous surgical history and new diagnosis or relapse cohort) Y/N

If N state what information/ test data you are aware of (e.g. barium FT)_____________________

**Segment**	**Quality of segmental visualisation to make correct diagnosis**<br><br>**1-good/ 2-moderate/ 3-poor/ 4-N/A or excised**				**If poor visualisation, tick why**<br><br>**1- poor distension/ *2- oral contrast not reached segment/ 3-Other (state)***		
	1	2	3	4	1	2	3
**duodenum**							
**Jejunum***							
**ileum**							
**Terminal ileum****							
**Caecum**							
**Ascending**							
**Transverse**							
**Descending**							
**Sigmoid**							
**Rectum**							

* small bowel from DJ flexure mainly to the left of a diagonal running from the RUQ to LLQ showing typical feathery fold pattern, ** last 10cm of ileum upstream of IV valve/anastomosis

UCL Clinical Trials Unit, Gower Street, London, WC1E 6BT
Tel: 020 3108 3263 Email: metric@ucl.ac.uk

PATIENT CASE REPORT FORM: MRI INTERPRETATION

Overall disease assessment (to be completed for all patients)						
	**Normal**		**Equivocal**		**Abnormal**	
**Confidence of presence**	1 (disease definitely not present)	2 (disease probably not present)	3 (disease possibly not present)	4 (disease possibly present)	5 (disease probably present)	6 (disease definitely present)
**Any small bowel disease PRESENT?-** tick confidence box						
**Confidence of activity**	1 (disease definitely not active)	2 (disease probably not active)	3 (disease possibly not active)	4 (disease possibly active)	5 (disease probably active)	6 (disease definitely active)
**If present (confidence score ≥3) is it ACTIVE?** tick confidence box						
**Confidence of presence**	1 (disease definitely not present)	2 (disease probably not present)	3 (disease possibly not present)	4 (disease possibly present)	5 (disease probably present)	6 (disease definitely present)
**Any colonic disease PRESENT?** tick confidence box						
**Confidence of activity**	1 (disease definitely not active)	2 (disease probably not active)	3 (disease possibly not active)	4 (disease possibly active)	5 (disease probably active)	6 (disease definitely active)
**If present (confidence score ≥3) is it ACTIVE?** tick confidence box						

UCL Clinical Trials Unit, Gower Street, London, WC1E 6BT

Tel: 020 3108 3263 Email: metric@ucl.ac.uk

METRIC

Patient Number ☐☐☐☐☐ Initials ☐☐☐

PATIENT CASE REPORT FORM: MRI INTERPRETATION

**Lymphadenopathy (0-3)**	☐ **0**	☐ **1**	☐ **2**	☐ **3**
**Abnormal free fluid (Y/N)**	☐ **Yes**		☐ **No**	
**Abscess present**	☐ **Yes**		☐ **No**	
If yes please state size & location				
**Fistula present (circle all that apply)**	☐ **Yes**		☐ **No**	
If yes please circle location		Ileo-ileal Ileo-colic entero-cutaneous ileo-vesical<br>colon-vesical jejuno- jejunal jejuno-colic<br>Other (state________________________________)		
**Other small bowel diagnosis (e.g. adhesions, meckels, radiation enteritis etc**	☐ **Yes**		☐ **No**	
**If yes please state**				
**Extra enteric findings (e.g. aortic aneurysm gallstones, solid organ abnormality, phelgmon)**	☐ **Yes**		☐ **No**	
If yes please state				
**Are you recommending any further tests?**	☐ **Yes**		☐ **No**	
If yes please state which				

PATIENT CASE REPORT FORM: MRI INTERPRETATION

Please complete for each segment	Confidence of disease PRESENCE					
	**Normal**		**Equivocal**		**Abnormal**	
**Segment**	1 (disease definitely not present)	2 (disease probably not present)	3 (disease possibly not present)	4 (disease possibly present)	5 (disease probably present)	6 (disease definitely present)
**Duodenum (D)**						
**Jejunum (J)**						
**Ileum (I)**						
**Terminal ileum (TI)***						
**Caecum (C)**						
**Ascending colon (A)**						
**Transverse colon (T)**						
**Descending colon (D)**						
**Sigmoid (S)**						
**Rectum (R)**						

*throughout, if TI disease is contiguous for over 10cm count just as TI not TI and ileum

Confidence of disease ACTIVITY **Please complete for each segment if confidence scores 3-6 for disease presence above i.e. present or equivocal**

		Normal		Equivocal		Active	
**Segment**	No disease (i.e. confidence scores 1 or 2 for disease presence)	1 (disease definitely not active)	2 (disease probably not active)	3 (disease possibly not active)	4 (disease possibly active)	5 (disease probably active)	6 (disease definitely active)
**Duodenum (D)**							
**Jejunum (J)**							
**Ileum (I)**							
**Terminal ileum (TI)**							
**Caecum (C)**							
**Ascending colon (A)**							
**Transverse colon (T)**							
**Descending colon (D)**							
**Sigmoid (S)**							
**Rectum (R)**							

PATIENT CASE REPORT FORM: MRI INTERPRETATION Disease Description **Please complete for each disease site (defined as >3cm of normal bowel between disease sites). Use one table for each disease site. Only record segments which if you have a confidence score of 3 or more for disease presence.** ***Use score definitions at the start of this CRF***

DISEASE SITE 1

Location	Tick one location	Single Wall thickness (mm) thickest portion	Wall thickening	Length of abnormal bowel (cm)	Stensois causing functional obstruction	Peri-mural T2 signal	Mural T2 signal	Ulceration	Contrast enhancement	Contrast enhancement pattern	Diffusion signal	Does the segment contain established fibrosis (Y/N)	Segmental disease severity assessment	Segment shows active disease (Y/N)
**Duo**														
**J**														
**I**														
**TI**														
**C**														
**A**														
**Des**														
**S**														
**R**														

PATIENT CASE REPORT FORM: MRI INTERPRETATION Disease Description Please complete for each disease site (defined as >3cm of normal bowel between disease sites). Use <u>one</u> table for each disease site. Only record segments which if you have a confidence score of 3 or more for disease presence. *Use score definitions at the start of this CRF*

DISEASE SITE 2

Location	Tick one location	Single Wall thickness (mm) thickest portion	Wall thickening	Length of abnormal bowel (cm)	Stensois causing functional obstruction	Peri-mural T2 signal	Mural T2 signal	Ulceration	Contrast enhancement	Contrast enhancement pattern	Diffusion signal	Does the segment contain established fibrosis (Y/N)	Segmental disease severity assessment	Segment shows active disease (Y/N)
**Duo**														
**J**														
**I**														
**TI**														
**C**														
**A**														
**Des**														
**S**														
**R**														

PATIENT CASE REPORT FORM: MRI INTERPRETATION Disease Description Please complete for each disease site (defined as >3cm of normal bowel between disease sites). Use one table for each disease site. Only record segments which if you have a confidence score of 3 or more for disease presence. *Use score definitions at the start of this CRF*

DISEASE SITE 3

Location	Tick one location	Single Wall thickness (mm) thickest portion	Wall thickening	Length of abnormal bowel (cm)	Stensois causing functional obstruction	Peri-mural T2 signal	Mural T2 signal	Ulceration	Contrast enhancement	Contrast enhancement pattern	Diffusion signal	Does the segment contain established fibrosis (Y/N)	Segmental disease severity assessment	Segment shows active disease (Y/N)
Duo														
J														
I														
TI														
C														
A														
Des														
S														
R														

PATIENT CASE REPORT FORM: MRI INTERPRETATION Disease Description Please complete for each disease site (defined as >3cm of normal bowel between disease sites). Use one table for each disease site. Only record segments which if you have a confidence score of 3 or more for disease presence. *Use score definitions at the start of this CRF*

DISEASE SITE 4

Location	Tick one location	Single Wall thickness (mm) thickest portion	Wall thickening	Length of abnormal bowel (cm)	Stensois causing functional obstruction	Peri-mural T2 signal	Mural T2 signal	Ulceration	Contrast enhancement	Contrast enhancement pattern	Diffusion signal	Does the segment contain established fibrosis (Y/N)	Segmental disease severity assessment	Segment shows active disease (Y/N)
Duo														
J														
I														
TI														
C														
A														
Des														
S														
R														

Please attach additional pages if required. If additional pages added, insert total number of additional pages used: _______________page(s)

Patient Number ☐☐☐☐☐ Initials ☐☐☐

PATIENT CASE REPORT FORM: MRI INTERPRETATION: Additional Sequences

	Diffusion weighted	Contrast enhanced
Not helpful	☐	☐
Diagnosis unchanged but increased confidence	☐	☐
Diagnosis changed-additional disease site detected	☐	☐
Diagnosis changed-disease site now discounted	☐	☐
Diagnosis changed –disease re-classified as active	☐	☐
Diagnosis changed –disease re-classified as inactive	☐	☐
Other-state		

Completed by:

Print name: *Signature:* *Date:*

Office Use Only:

Received by (Print name & sign): *Date:* *Entered by: (Print name & sign):* *Date:*
