# Supplementary material for: Diagnostic Performance of Magnetic Resonance Enterography Disease Activity Indices Compared with a Histological Reference Standard for Adult Terminal Ileal Crohn’s Disease: Experience from the METRIC Trial
Source: J Crohns Colitis. 2022 Jun 8;16(10):1531–9. doi: 10.1093/ecco-jcc/jjac062 (PMC9624291; doi:10.1093/ecco-jcc/jjac062)
Supplement: jjac062_suppl_Supplementary_Appendix_2 [file jjac062_suppl_supplementary_appendix_2.docx]

**Appendix 2.** Definitions of the sMARIA, London and “extended” London scores.

**sMARIA = (1 x wall thickness > 3 mm) + (1 x wall oedema) + (1 x fat stranding) + (2 x ulcers)**

Wall thickness >3mm scores 1 point

Presence of oedema scores 1 point

Presence of fat stranding scores 1 point (we used perimural stranding as a surrogate for this)

Presence of ulcers scores 2 points

Thus, the score for each segment ranges from 0 to 5.

**London = 1.79 + (1.34 x mural thickness) + (0.94 x mural T2 score)**

**Extended London = mural thickness + mural T2 score + perimural T2 signal + contrast enhancement**

| **Score** | **0** | **1** | **2** | **3** |
| --- | --- | --- | --- | --- |
| **Mural thickness^1^** | 1–3mm | >3–5mm | >5–7mm | >7mm |
| **Mural T2 signal^2^** | Equivalent to normal bowel  wall | Minor increase in signal-bowel  wall appears dark grey on fat  saturated images | Moderate increase in  signal-bowel wall appears light  grey on fat saturated images | Marked increase in  signal-bowel wall contains  areas of white high signal  approaching that of luminal  content |
| **Perimural T2 signal** | Equivalent to normal  mesentery | Increase in mesenteric signal  but no fluid | Small fluid rim (≤2mm) | Larger fluid rim (>2mm) |
| **Enhancement^3^** | Equivalent to normal bowel  wall | Minor enhancement – bowel  wall signal greater than normal  small bowel but significantly  less than nearby vascular  structures | Moderate enhancement –  bowel wall signal increased but  somewhat less than nearby vascular  structures | Marked enhancement – bowel  wall signal approaches that of  nearby vascular structures |

^1^ Measured using electronic calipers

^2^ Compared to normal small bowel
