# Supplementary figures and images for: Diagnostic Performance of Magnetic Resonance Enterography Disease Activity Indices Compared with a Histological Reference Standard for Adult Terminal Ileal Crohn’s Disease: Experience from the METRIC Trial
Source: J Crohns Colitis. 2022 Jun 8;16(10):1531–9. doi: 10.1093/ecco-jcc/jjac062 (PMC9624291; doi:10.1093/ecco-jcc/jjac062)

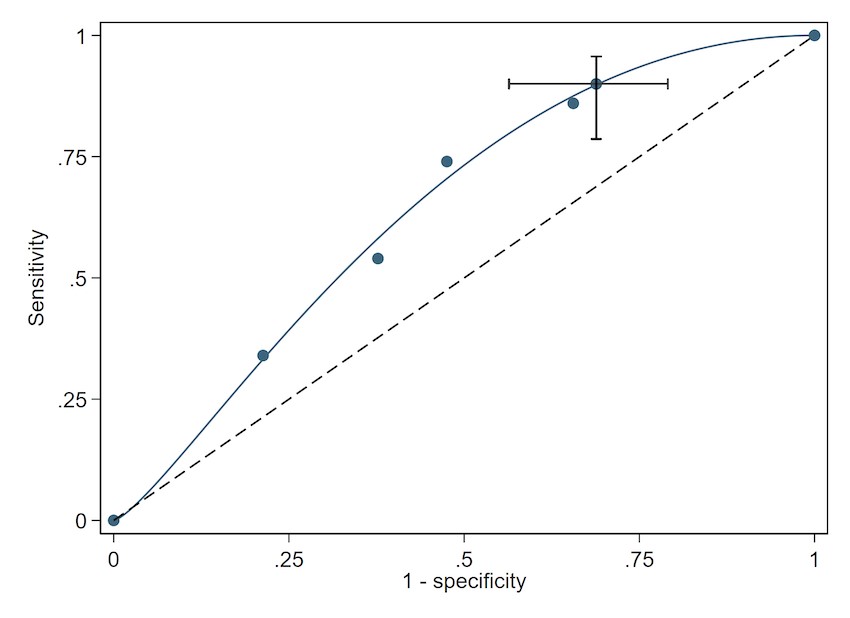

Supplement: jjac062_suppl_Supplementary_Appendix_3a [file jjac062_suppl_supplementary_appendix_3a.jpeg]

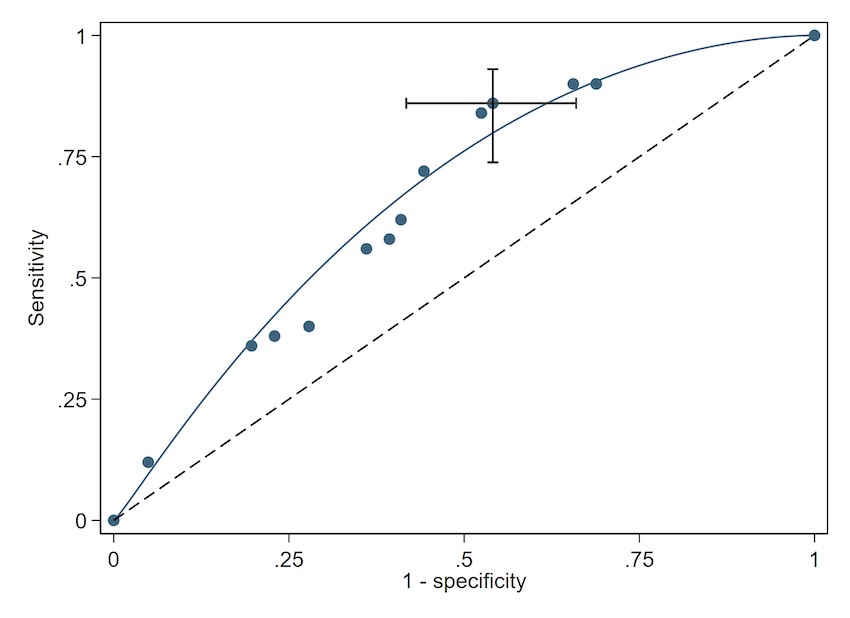

Supplement: jjac062_suppl_Supplementary_Appendix_3b [file jjac062_suppl_supplementary_appendix_3b.jpeg]

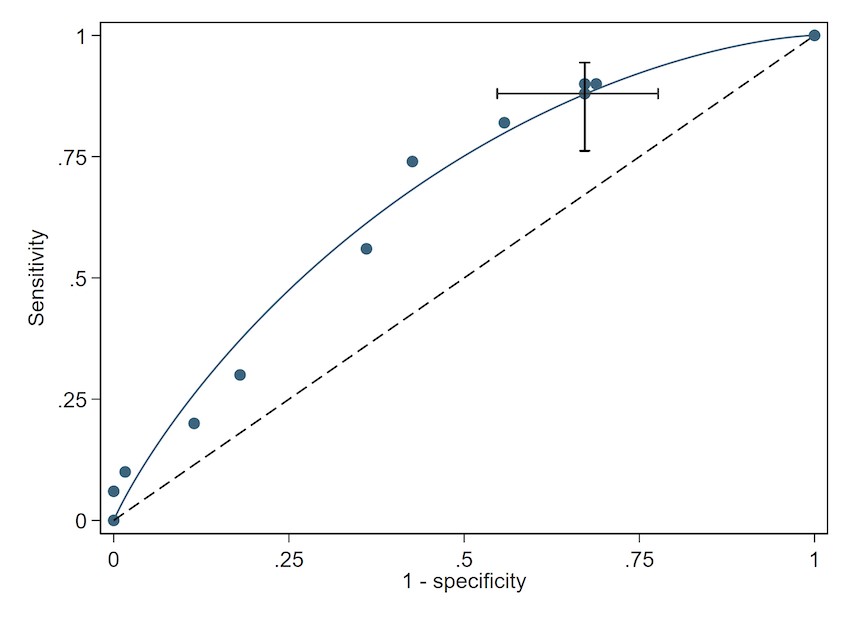

Supplement: jjac062_suppl_Supplementary_Appendix_3c [file jjac062_suppl_supplementary_appendix_3c.jpeg]

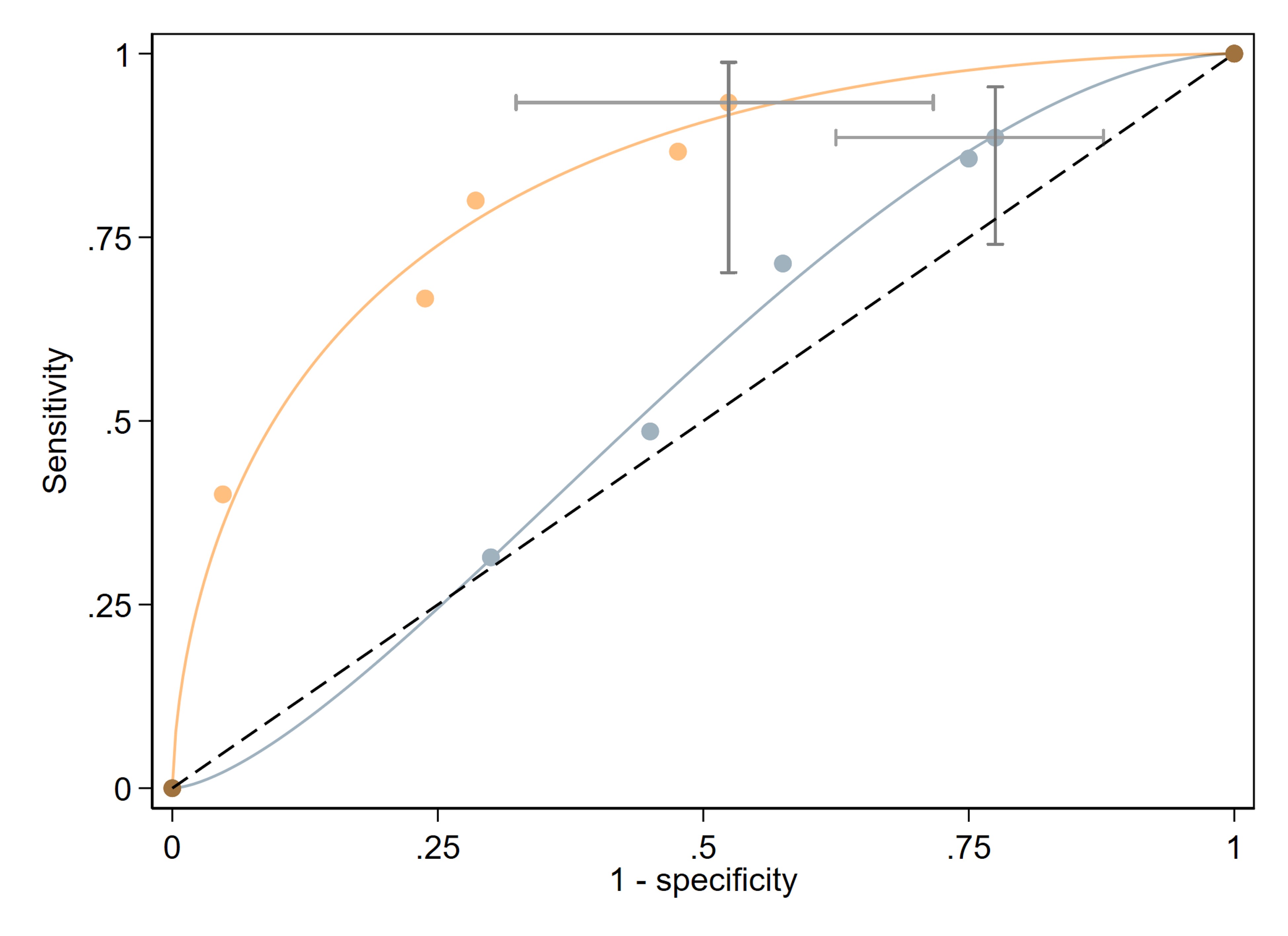

Supplement: jjac062_suppl_Supplementary_Appendix_4a [file jjac062_suppl_supplementary_appendix_4a.jpeg]

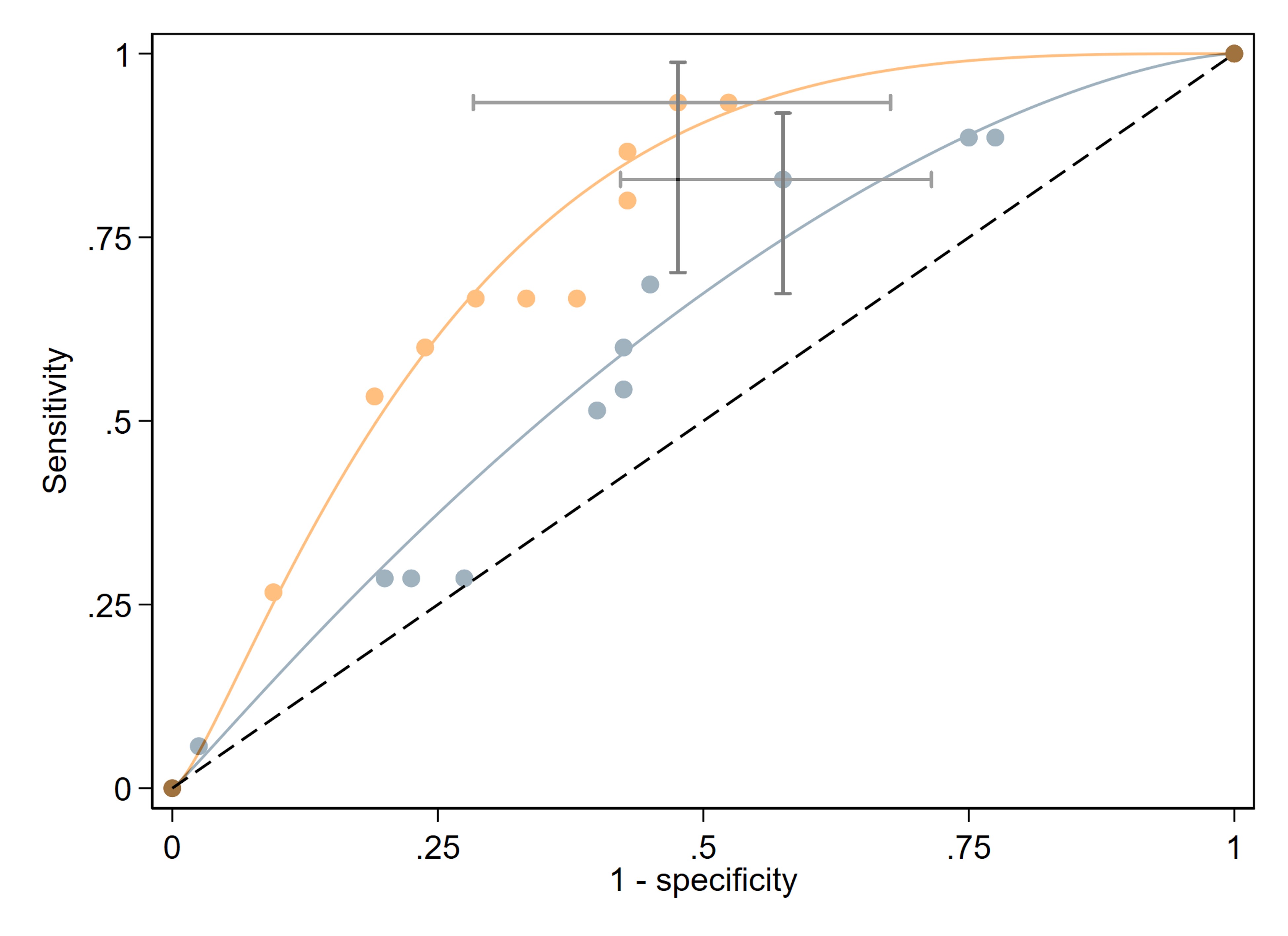

Supplement: jjac062_suppl_Supplementary_Appendix_4b [file jjac062_suppl_supplementary_appendix_4b.jpeg]

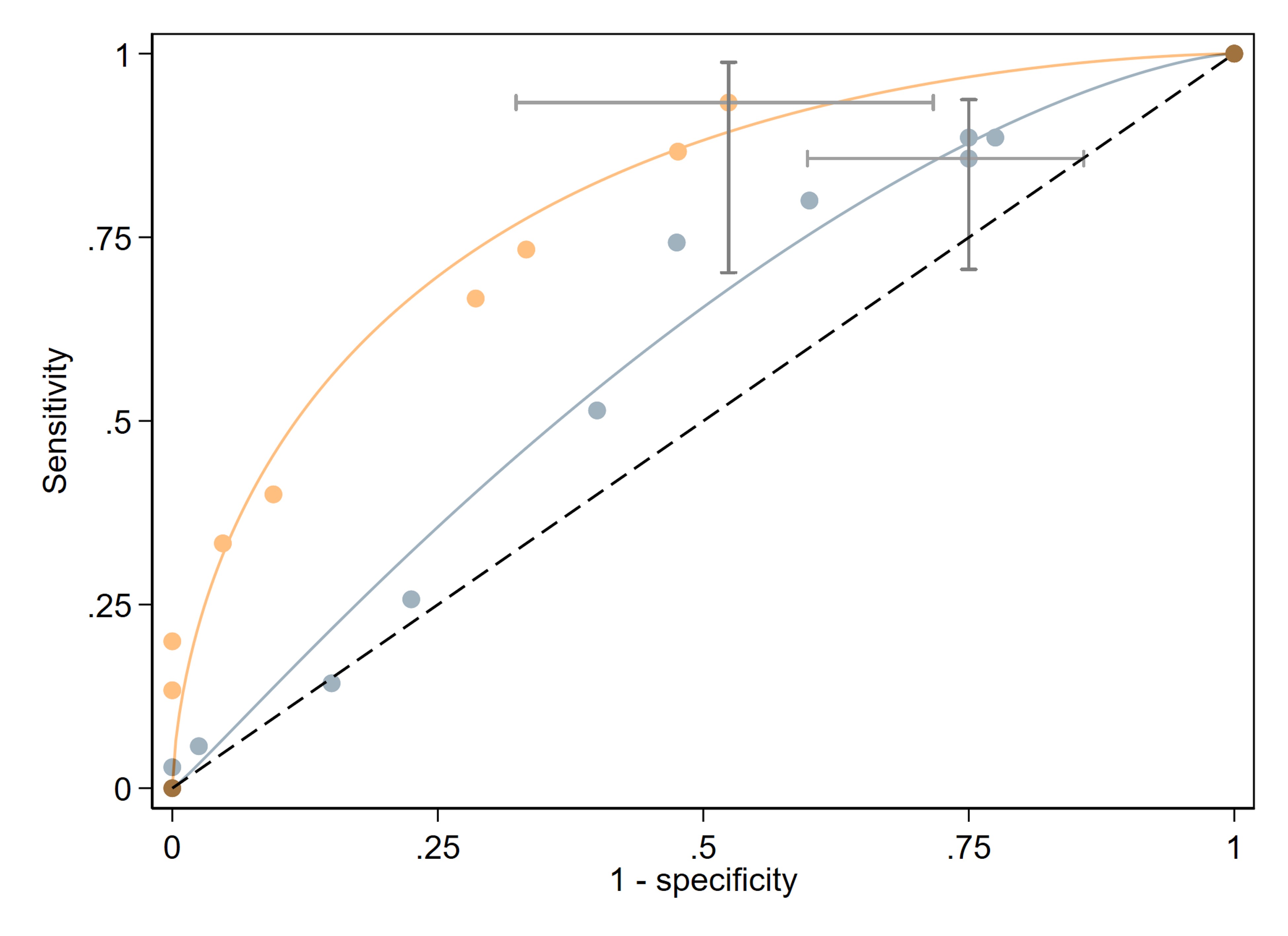

Supplement: jjac062_suppl_Supplementary_Appendix_4c [file jjac062_suppl_supplementary_appendix_4c.jpeg]
