# Supplementary material for: Diagnostic Performance of Magnetic Resonance Enterography Disease Activity Indices Compared with a Histological Reference Standard for Adult Terminal Ileal Crohn’s Disease: Experience from the METRIC Trial
Source: J Crohns Colitis. 2022 Jun 8;16(10):1531–9. doi: 10.1093/ecco-jcc/jjac062 (PMC9624291; doi:10.1093/ecco-jcc/jjac062)
Supplement: jjac062_suppl_Supplementary_Tables [file jjac062_suppl_supplementary_tables.docx]

| **Inflammatory Activity** | **Score** | **Histopathologic Defining Characteristics** |
| --- | --- | --- |
| Inactive/ Quiescent/ Normal | 0 | No epithelial infiltration by neutrophils. |
| Mildly Active | 1 | Neutrophil infiltration of <50% of sampled crypts or cross-sections, no ulcers, or erosions. |
| Moderately Active | 2 | Neutrophil infiltration of ≥ 50% of sampled crypts or cross-sections, no ulcers, or erosions. |
| Severely Active | 3 | Erosion or ulceration, irrespective of other features. |

a. Inactive colitis with no cryptitis or crypt abscesses; HAI=0.

b. Mildly active colitis with one crypt abscess (arrow); HAI=1

c. Moderately active colitis with cryptitis involving >50% of crypts (arrows); HAI=2.

d. Severely active colitis with ulceration; HAI=3.

**Supplemental Table 1.** Histological Activity Index (HAI)

|  | **All patients**  **N (%)** | **Newly diagnosed**  **N (%)** | **Suspected relapse**  **N (%)** |
| --- | --- | --- | --- |
| **0** | 24 (22) | 13 (17) | 11 (31) |
| **1** | 4 (4) | 2 (3) | 2 (6) |
| **2** | 17 (15) | 12 (16) | 5 (14) |
| **3** | 16 (14) | 13 (17) | 3 (8) |
| **4** | 20 (18) | 12 (16) | 8 (22) |
| **5** | 30 (27) | 23 (31) | 7 (19) |

**Supplemental Table 2.** Tabulation of sMARIA scores in the study population.

|  | **All patients** | **Newly diagnosed** | **Suspected relapse** |
| --- | --- | --- | --- |
| **Median** | 6 | 6 | 6 |
| **Lower IQR** | 4 | 4 | 2 |
| **Upper IQR** | 8 | 7 | 8 |

**Supplemental Table 3.** Tabulation of London scores in the study population. IQR = interquartile range.

|  | **All patients**  **N (%)** | **Newly diagnosed**  **N (%)** | **Suspected relapse**  **N (%)** |
| --- | --- | --- | --- |
| **0** | 24 (22) | 13 (17) | 11 (31) |
| **1** | 1 (1) | 1 (1) | 0 (0) |
| **2** | 1 (1) | 1 (1) | 0 (0) |
| **3** | 10 (9) | 8 (11) | 2 (6) |
| **4** | 12 (11) | 7 (9) | 5 (14) |
| **5** | 13 (12) | 11 (15) | 2 (6) |
| **6** | 24 (22) | 16 (21) | 8 (22) |
| **7** | 9 (8) | 7 (9) | 2 (6) |
| **8** | 11 (10) | 8 (11) | 3 (8) |
| **9** | 3 (3) | 2 (3) | 1 (3) |
| **10** | 3 (3) | 1 (1) | 2 (6) |

**Supplemental Table 4.** Tabulation of “extended” London scores in the study population.

| **Recruitment institution** | **All patients**  **N (%)** | **Newly diagnosed**  **N (%)** | **Suspected relapse**  **N (%)** |
| --- | --- | --- | --- |
| Site 1 | 16 (14) | 12 (16) | 4 (11) |
| Site 2 | 13 (12) | 7 (9) | 6 (17) |
| Site 3 | 13 (12) | 7 (9) | 6 (17) |
| Site 4 | 15 (14) | 12 (16) | 3 (8) |
| Site 5 | 3 (3) | 1 (1) | 2 (6) |
| Site 6 | 3 (3) | 2 (3) | 1 (3) |
| Site 7 | 48 (43) | 34 (45) | 14 (39) |

**Supplemental Table 5.** Patients split by recruiting site.

| **Variable** | **sMARIA <1**  **n = 9** | **sMARIA >=1**  **n = 13** | **p-value** |
| --- | --- | --- | --- |
| **Age (years)** | | | |
| Median (IQR) | 34 (30 to 45) | 29 (20 to 35) | 0.15 |
| **Sex,** **no. (%)** 0.78 | | | |
| Female | 5 (56) | 8 (62) | - |
| Male | 4 (44) | 5 (38) | - |
| **HBI** |  | | |
| Median (IQR) | 6 (0.5 to 10) | 5 (2 to 7) | 0.89 |
| **EQ-5D** | | | |
| Median (IQR) | 80 (75 to 90) | 70 (35 to 85) | 0.23 |
| **CRP (mg/L)** |  |  |  |
| Median (IQR) | 2.9 (0.2 to 9.7) | 6.3 (2.3 to 21) | 0.15 |
| **Calprotectin (μg/g)** |  |  |  |
| Median (IQR) | 419 (60 to 600) | 686 (360 to 1360) | 0.11 |

**Supplemental Table 6.** Clinical characteristics for patients with normal histology of the terminal ileum (HAI = 1) stratified by inactive and active disease according to sMARIA. sMARIA = simplified magnetic resonance enterography index. IQR = interquartile range. HBI = Harvey-Bradshaw Index. EQ-5D = EuroQol five-dimension questionnaire. CRP = C-reactive protein.

| **Variable** | **London <4.1**  **n = 14** | **London >=4.1**  **n = 8** | **p-value** |
| --- | --- | --- | --- |
| **Age (years)** | | | |
| Median (IQR) | 31.5 (22 to 45) | 29 (22 to 38.5) | 0.56 |
| **Sex,** **no. (%)** 0.51 | | | |
| Female | 9 (64) | 4 (50) | - |
| Male | 5 (36) | 4 (50) | - |
| **HBI** |  | | |
| Median (IQR) | 5 (0.7 to 8.5) | 6 (2 to 8) | 0.60 |
| **EQ-5D** | | | |
| Median (IQR) | 80 (60 to 90) | 77.5 (50 to 90) | 0.83 |
| **CRP (mg/L)** |  |  |  |
| Median (IQR) | 3.2 (1.3 to 7.4) | 17.8 (4.2 to 68) | 0.076 |
| **Calprotectin (μg/g)** |  |  |  |
| Median (IQR) | 419 (120.2 to 600) | 686 (482 to 1263) | 0.26 |

**Supplemental Table 7.** Clinical characteristics for patients with normal histology of the terminal ileum (HAI = 1) stratified by inactive and active disease according to the London score. IQR = interquartile range. HBI = Harvey-Bradshaw Index. EQ-5D = EuroQol five-dimension questionnaire. CRP = C-reactive protein.

| **Variable** | **“Extended” London <3**  **n = 9** | **“Extended” London >=3**  **n = 13** | **p-value** |
| --- | --- | --- | --- |
| **Age (years)** | | | |
| Median (IQR) | 34 (30 to 45) | 29 (20 to 35) | 0.15 |
| **Sex,** **no. (%)** 0.78 | | | |
| Female | 5 (56) | 8 (62) | - |
| Male | 4 (44) | 5 (38) | - |
| **HBI** |  | | |
| Median (IQR) | 6 (0.5 to 10) | 5 (2 to 7) | 0.89 |
| **EQ-5D** | | | |
| Median (IQR) | 80 (75 to 90) | 70 (35 to 85) | 0.23 |
| **CRP (mg/L)** |  |  |  |
| Median (IQR) | 2.9 (0.2 to 9.7) | 6.3 (2.3 to 21) | 0.15 |
| **Calprotectin (μg/g)** |  |  |  |
| Median (IQR) | 419 (60 to 600) | 686 (360 to 1360) | 0.11 |

**Supplemental Table 8.** Clinical characteristics for patients with normal histology of the terminal ileum (HAI = 1) stratified by inactive and active disease according to the “extended” London score. IQR = interquartile range. HBI = Harvey-Bradshaw Index. EQ-5D = EuroQol five-dimension questionnaire. CRP = C-reactive protein.

**Appendices**

**Appendix 1.** Standardised clinical report form (CRF) documenting conventional MRE observations

**Appendix 2.** Definitions of the sMARIA, London and “extended” London scores.

**Appendix 3.** ROC curves for detecting active disease (HAI > 1) for (a) sMARIA, (b) London, and (c) “extended” London score. Grey bars report 95% confidence intervals at the pre-specified thresholds.

**Appendix 4.** ROC curves for detecting active disease (HAI > 1) stratified by newly diagnosed and suspected relapse patients for (a) sMARIA, (b) London, and (c) “extended” London scores. Grey bars report 95% confidence intervals at the pre-specified thresholds.
